# Volatile unsaturated hydrocarbons emitted by seedlings of *Brassica* species provide host location cues to *Bagrada hilaris*

**DOI:** 10.1371/journal.pone.0209870

**Published:** 2018-12-27

**Authors:** Salvatore Guarino, Mokhtar Abdulsattar Arif, Jocelyn G. Millar, Stefano Colazza, Ezio Peri

**Affiliations:** 1 Dipartimento di Scienze Agrarie, Alimentari e Forestali, Università degli Studi di Palermo, Palermo, Italy; 2 Department of Entomology, University of California, Riverside, California, United States of America; University of Catania, ITALY

## Abstract

*Bagrada hilaris* Burmeister, is a stink bug native to Asia and Africa and invasive in the United States, Mexico, and more recently, South America. This species can cause serious damage to various vegetable crops in the genus *Brassica*, with seedlings being particularly susceptible to *B*. *hilaris* feeding activity. In this study, the role of volatile organic compounds (VOCs) emitted by seedlings of three *Brassica* species on the host preference of *B*. *hilaris* was evaluated. In dual choice arena and olfactometer bioassays, adult painted bugs preferred *B*. *oleracea* var. botrytis and *B*. *napus* over *B*. *carinata*. Volatiles from *B*. *oleracea* seedlings were collected and bioassayed with *B*. *hilaris* adults and late stage nymphs, using electroantennographic (EAG) and behavioral (olfactometer) techniques. When crude extracts of the VOCs from *B*. *oleracea* var. botrytis seedlings and liquid chromatography fractions thereof were bioassayed, *B*. *hilaris* adults and nymphs were attracted to the crude extract, and to a non-polar fraction containing hydrocarbons, whereas there were no responses to the more polar fractions. GC-MS analysis indicated that the main constituents of the non-polar fraction was an as yet unidentified diterpene hydrocarbon, with trace amounts of several other diterpene hydrocarbons. The major diterpene occurred in VOCs from both of the preferred host plants *B*. *oleracea* and *B*. *napus*, but not in VOCs of *B*. *carinata*. Our results suggest that this diterpene, alone or in combination with one or more of the minor compounds, is a key mediator in this insect-plant interaction, and could be a good candidate for use in lures for monitoring *B*. *hilaris* in the field.

## Introduction

The painted bug, *Bagrada hilaris* Burmeister, is an invasive stink bug which feeds mainly on brassicaceous hosts, and it seems particularly damaging to crops in recently invaded areas [1]. This species is widely distributed across Africa and southern Europe, and east through Pakistan, India, China, and parts of southeast Asia [2, 3]. In 2008, the painted bug was first reported in California, probably introduced by commercial trade, and it rapidly expanded its range to the brassicaceous crops of coastal California and central Arizona [4], and then to Nevada, New Mexico, and Utah [5]. More recently, *B*. *hilaris* has been reported from Mexico [6], from the island of Maui in Hawaii [7], and in Chile [8]. *Bagrada hilaris* has had a major impact on agriculture in the Americas; it has been estimated that about 90% of the broccoli acreage planted in the USA has been infested by the painted bug, with yield losses often exceeding 10% of production [1]. A recent study by Guarino et al. [9] showed that infestation of a cauliflower plant, *Brassica oleracea* var. botrytis L., by 40 adult painted bugs reduced leaf photosynthesis by about 40%. To date, effective sampling strategies and good monitoring tools for this pest, for example based on semiochemicals, have not yet been developed [3]. Other stink bug species are currently monitored with traps baited with sex or aggregation pheromones [10–13]. In the case of *B*. *hilaris*, (*E*)-2-octenyl acetate was reported as a possible pheromone for this species [14], but attempts to develop this compound into a reliable monitoring tool have not yet been successful [3]. Consequently, alternative lures based on host plant volatile organic compounds (VOCs) may be good candidates for use in monitoring traps. Host plant VOCs are known to be exploited by phytophagous pentatomid species as host location cues [15, 16]. However, information about the VOCs that might serve as host attractants for *B*. *hilaris* is limited [16]. Recent electroantennographic studies showed that adults of this species perceive several plant VOCs, including octanal, nonanal, acetic acid, benzaldehyde, 3-butenyl isothiocyanate, and 4-pentenyl isothiocyanate [7, 9]. Among these chemicals, only isothiocyanates are characteristic to *Brassica* spp., and they are produced mainly upon damage to tissues rather than by undamaged plants [9, 17, 18]. In field bioassays, *B*. *hilaris* were not consistently attracted to traps baited with various isothiocyanates, either singly or in blends [7], and to date, no consistently effective plant-derived attractants have been identified for this species [3]. Any search for candidate attractants for herbivorous pests should consider not only the preferred host plant species and their main VOCs, but also other plant traits, which may be indicators of host quality as food and/or shelter. These characteristics may change during plant development, and as a consequence, the attraction of *B*. *hilaris* may change with the development of its hosts [19].

In contrast to other stink bug specialists of Brassicaceae, such as *Murgantia histrionica* (Hahn), which prefers to feed on plants with at least 4–5 pairs of true leaves [20, 21], the painted bug appears to strongly prefer host plants at the stage of newly emerged seedlings [1, 22]. The feeding damage of *B*. *hilaris* on cotyledons and young tissues results in significant reductions in leaf area, chlorophyll content, and dry weight, if it does not kill the seedling outright [1, 6]. This suggests that VOCs produced by seedlings of preferred *B*. *hilaris* host plants are likely to be particularly attractive to the bugs. Further clues as to the identities of host-produced attractants can be obtained by comparing the profiles of VOCs produced by more or less susceptible brassicaceous species and varieties. The identification of these VOCs could have several major benefits. First, it will lead to a better understanding of the ecology of *B*. *hilaris*, and the factors mediating its host selection behaviors. Second, given the demonstrated strong attraction of *B*. *hilaris* to *Brassica* seedlings, the attractant chemicals would be excellent candidates for development into tools for monitoring and attract-and-kill strategies. Third, identification of the attractants could inform traditional or molecular biological plant breeding efforts to eliminate the attractants from the host plant VOCs. Thus, the goal of this study was to characterize the volatile cue(s) exploited during host location by *B*. *hilaris*. Our specific objectives were:

1. Among possible host plants in the *Brassica* genus, to identify species that differed in attractiveness at the seedling stage to *B*. *hilaris* nymphs and adults;
2. To prepare active extracts of one or more attractive species;
3. To use bioassay-driven fractionation to characterize the attractant(s) present in extracts from the attractive species.

We report here the characterization of a diterpene hydrocarbon as a key host plant attractant for *B*. *hilaris*.

## Material and methods

### Insects

The colony of *B*. *hilaris* was established and restocked regularly with individuals collected from caper (*Capparis spinosa* L.) fields on the island of Pantelleria (Italy), with permission from the owner of the land. Insects were reared in an environmentally controlled room (30 ± 2°C, 70 ± 10% RH, photoperiod 16L:8D), in wooden cages (40 x 25 × 25 × 40 cm) with two 5-cm diameter mesh-covered holes for ventilation. The colony was fed with cauliflower and cabbage plants, depending on seasonal availability. Because *B*. *hilaris* lays eggs in the soil, dishes (6-cm Ø) with a mixture of sand, silt, and clay (33% for each soil component) were placed in the cages as oviposition sites. Dishes were changed weekly, and those with eggs were kept in separate cages until the emergence of nymphs. The nymphs were then kept in separate cages until the final molt to adults. Adults and 4^th^-5^th^ instar nymphs were used separately in experiments.

### Seedlings

Seeds of *B*. *oleracea* var. botrytis (cauliflower), *B*. *napus* (rapeseed), and *B*. *carinata* (Abyssinian cabbage), all obtained from a local market (Palermo–Italy), were placed on cotton wool (10 g) soaked with distilled water and held in glass containers with a distance of circa 0.5 cm between seeds. The containers were placed in an environmentally controlled growth chamber (25 ± 1°C, 70 ± 10% RH, photoperiod 16L:8D) equipped with lights with a photosynthetic flux density (PPFD) of 600 mol photons m^-2^ s^-1^ placed above the foliage. To avoid desiccation, soaked seeds were covered with a Petri dish for 3 d until they germinated. After 7 d, newly emerged seedling clusters at the cotyledon stage were wrapped around the base with aluminum foil for use in host preference bioassays, VOC collection, or behavioral experiments.

### Dual choice arena

The dual choice arena consisted of a Plexiglas cage 50 cm wide, 30 cm high, and 30 cm deep with mesh for ventilation on opposing sides. Inside the cage, two clusters of 7-d old seedlings of the different plant varieties (N = 20) were placed 20 cm apart. All three possible combinations of pairs of the three plant species were tested. Bioassays were carried out in static air, using in parallel 6 cages for each session of experiments. Adult bugs were released individually into each cage at 2:00 PM for each replicate and as a response the presence of the bug in one of the seedlings cluster was recorded after 20h, at 10:00 AM the next day (final choice). The position of the treatments was alternated between replicates. After each replicate, the Plexiglas cage was cleaned with water and dried. Bioassays were conducted under ambient laboratory temperature and humidity conditions (25 ± 3°C, and 50 ± 15% RH).

### Open vertical Y-shaped olfactometer

Further bioassays were carried out with an open vertical Y-shaped olfactometer consisting of three connected brass rods (left and right arms 20 cm long, central arm 25 cm long, 1 cm diameter). The left and right arms were covered with two glass tubes (18 cm long, 5 cm diameter) terminating in hose nipples. Charcoal-purified air (0.2 l/min per chamber) was directed through two glass chambers (125 ml each), which held the test stimuli, consisting of seedling clusters (N = 50), or extracts of headspace volatiles released by seedlings (see below). The outlets of the stimulus chambers were connected to the hose nipples on the respective arms of the olfactometer with Tygon tubing. Light was provided with a halogen lamp (Osram, 12V–35W, Münich, Germany) hanging 30 cm above the olfactometer. Experiments were carried out under ambient laboratory temperature and humidity conditions (25 ± 3°C, and 50± 15% RH). For each replicate, a single adult or nymph was gently placed at the bottom of the central arm of the olfactometer with a paintbrush and allowed 10 min to respond. The bugs moved from the bottom upward toward the light source and upon arriving at the Y junction, choose between the two different volatile stimuli. The criterion for a response was that the test bug walked onto the test arm or the control arm at least 5 cm past the Y junction (first choice). Bugs that did not move onto one of the two arms during the 10 min trial were scored as non-responders and were not included in the analysis. After 8 replicates, the glass parts of the apparatus were washed with water and detergent, and then wiped with acetone and the brass rods were cleaned with distilled water and acetone and baked at 200°C for 60 min.

### Electroantennography

Electroantennograms (EAG) were conducted using an aliquot of 2 μl of each test or control stimulus solution pipetted onto a piece of filter paper (Whatman, grade 1). The loaded paper was exposed to the air for 30s to allow the solvent to evaporate, and then inserted into a glass Pasteur pipette. Puff stimuli were blown into an airstream that passed over the antennal preparation using a flow controller (model CS-05; Syntech, Hilversum, the Netherlands) to generate a 1.5-s stimulus at 1-min intervals, with a flow rate of 1.5 l min^−1^. The signals generated by the antennae were passed through a high-impedance amplifier (model IDAC-4, Syntech) and recorded with custom software (Syntech). For the EAG preparations, adult and nymphs *B*. *hilaris* were anesthetized by refrigerating them at about −4°C for 40s, the head was excised and mounted on a glass capillary reference electrode (1.5 mm diameter) filled with 0.1 M KCl solution and connected with silver wire to the amplifier. The recording electrode was a similar glass capillary in contact with the tip of an antenna. The capillary tubes were drawn to a fine point using a microelectrode puller (Narishige PC-10, Tokyo, Japan) to achieve a diameter enabling insertion into the antennal tip.

### VOC collection and fractionation

VOC collections were carried out using clusters of 7-d-old seedlings (N = 500) of *B*. *oleracea* var. botritys, *B*. *carinata*, and *B*. *napus*. Seedling clusters were placed in a cylindrical glass chamber (3 l volume) and a stream of air purified by passing through a filter of activated charcoal (0.5–1.0 mm, 18–35 mesh, Merck, Darmstadt, Germany), was pumped through the chamber at 400 ml/min. A glass tube containing a plug of 100 mg of Porapak Q (80–100 mesh; Sigma-Aldrich) was used to collect the VOCs. After collecting for 20 h, the traps were eluted with 1 ml of hexane, and the resulting extracts were concentrated to ~100 μl under a gentle nitrogen stream. Extracts were stored at −20°C in glass vials with Teflon cap liners until used for (GC/MS) analyses and bioassay experiments. All replicates were carried out in a temperature-controlled room (25 ± 3°C, 60 ± 5% RH and photoperiod 16L: 8D). After each collection, the chamber was washed with water and fragrance-free detergent, rinsed with acetone, and baked overnight at 150°C. Blank aerations were carried out as controls to identify possible system contaminants.

Aliquots of 1 ml of VOC extracts from seedlings (ten aerations pooled together) were fractionated by liquid chromatography into non-polar and polar fractions by using a solid phase extraction cartridge (SPE, silica column DSC-SI 52652-U, 1 ml tube, Supelco). The silica gel cartridge was conditioned by rinsing with 1 ml of hexane. The extract was loaded onto the cartridge as a hexane solution, followed by elution with 1 ml of hexane to obtain a non-polar hydrocarbons fraction. The cartridge then was eluted with 1 ml of dichloromethane and 1 ml of ethyl alcohol to extract the medium and high polarity compounds respectively. The fractions were kept at -20 C° until used for GC/MS analyses or electrophysiological and behavioral bioassays.

### Behavioral bioassays

#### Host preference bioassays

This experiment was carried out to assess the host preference of *B*. *hilaris* adults (sex ratio 1:1) to different potential host plants in two-choice tests using both the dual choice arena and the open vertical Y-shaped olfactometer. The experimental design was: *B*. *oleracea* var. botritys vs *B*. *carinata*, *B*. *napus* vs *B*. *carinata*, *B*. *oleracea* var. botritys vs *B*. *napus*. The number of replicates for each experiment was respectively 88, 85 and 106 in the dual choice arena and 120 in the open vertical Y-shaped olfactometer.

#### Bioassays with *B. oleracea* var. botrytis VOCs

In this experiment, volatiles from *B*. *oleracea* var. botrytis were bioassayed with adults and nymphs using EAG recording and open vertical Y-shaped olfactometer tests. EAG experiments tested 2 μl of the VOC crude extract, corresponding to approximately 200 seedling-hours, as a stimulus, versus solvent controls (hexane) (N = 20 nymphs; N = 15 adults). In a second experiment, responses elicited by the non-polar, medium polarity, and high polarity fractions were compared, with values normalized by subtracting the response elicited from the respective solvent control (hexane, dichloromethane, or ethyl alcohol) (N = 20 nymphs; N = 18 adults).

In the Y-shaped olfactometer, VOCs emitted from *B*. *oleracea* var. botrytis were tested according to the following design: 1) seedlings of *B*. *oleracea* var. botrytis (test) vs air (control) (N = 116 nymphs; N = 133 adults); 2) VOC extracts, corresponding to 200 seedling-hours, (test) vs hexane (control) (N = 188 nymphs; N = 200 adults); 3) Non-polar fraction (test) vs hexane (control) (N = 159 nymphs; N = 164 adults; 4) Medium polarity fraction (test) vs dichloromethane (control) (N = 161 nymphs; N = 169 adults); 5) Highly polar fraction (test) vs ethyl alcohol (control) (N = 160 nymphs; N = 160 adults).

### Chemical analysis

Coupled gas chromatography-mass spectrometry (GC-MS) analyses of the headspace extracts from *B*. *oleracea* var. botrytis, *B*. *napus*, and *B*. *carinata* were performed on an Agilent 6890 GC system interfaced with an MS5973 quadruple mass spectrometer. One μl of extract was injected onto a DB5-MS column in splitless mode. Injector and detector temperatures were 260°C and 280°C respectively. Helium was used as the carrier gas. The GC oven temperature was set at 40°C for 5 min, then increased by 10°C/min to 250°C. Electron impact ionization spectra were obtained at 70 eV, recording mass spectra from 40 to 550 amu. Four replicates were carried out for each plant species.

An aliquot of a VOC extract in hexane was reduced by stirring with 5% Pd on charcoal catalyst under a hydrogen atmosphere for 1 h. After filtration through a plug of celite to remove the catalyst, the solution was analyzed by GC-MS as described above.

### Statistical analysis

Mean values of the antennal depolarization responses elicited by the various test stimuli in EAG trials with *B*. *hilaris* antennae were prior examined for normality distribution by Shapiro-Wilk test and then analyzed by one-way ANOVA followed by Tukey’s test. Data from dual choice arena and open vertical Y-shaped olfactometer experiments, respectively, comparing the final and the first choice between the test or control, were analyzed with *χ*^2^ tests. All the statistical analyses were performed using Statistica 7.0 for Window (Statsoft 2001, Vigonza, PD, Italy).

## Results

### Host preference bioassays

**Fig 1:**
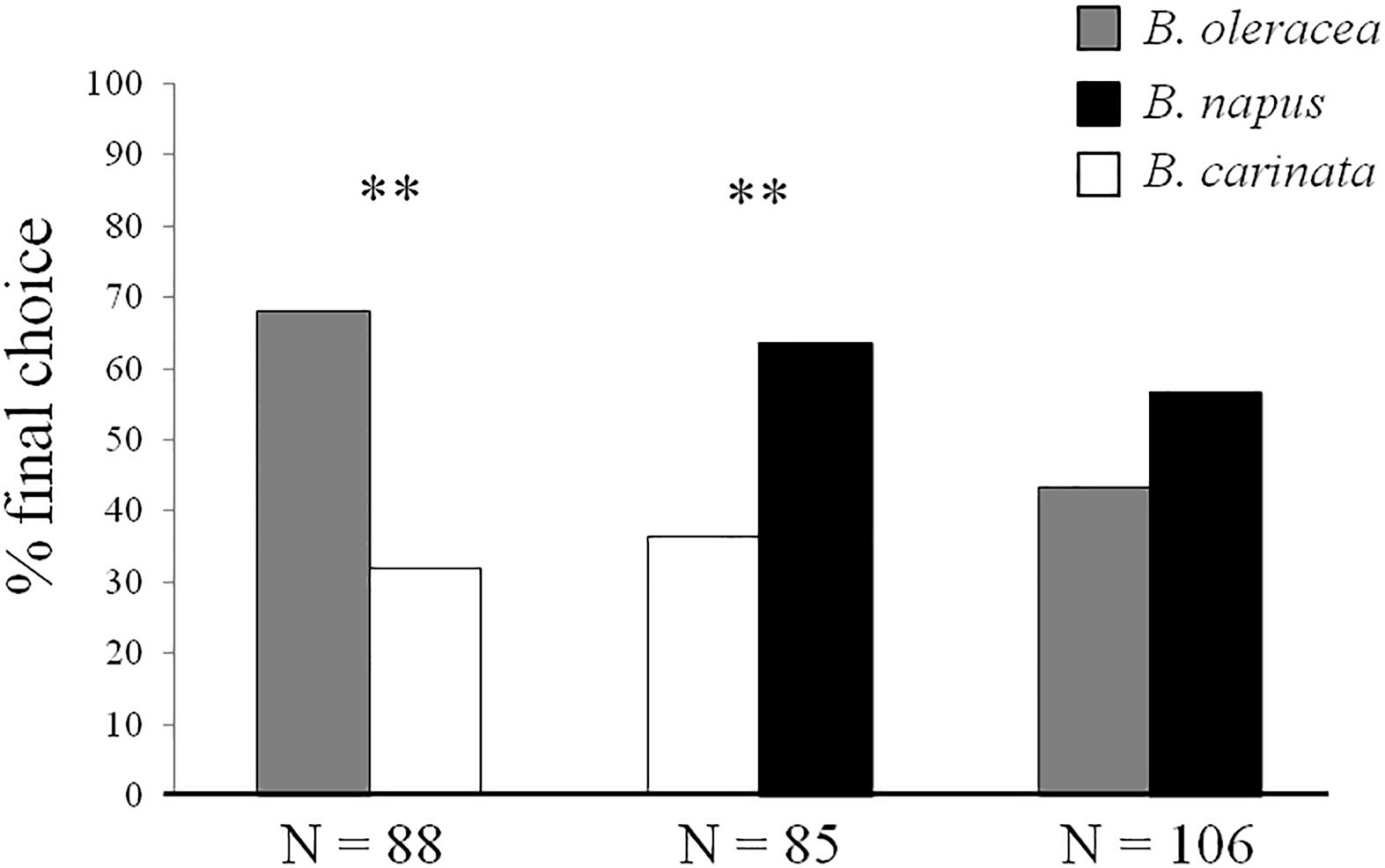
Dual choice arena bioassays. Host preference responses (% final choice) of *B*. *hilaris* adults to seedlings of *B*. *oleracea* var. botrytis, *B*. *carinata*, and *B*. *napus*. N = number of replicates; ** = P < 0.01; *χ*^2^.

In dual choice arena bioassays (Fig 1), *B*. *hilaris* adults preferred *B*. *oleracea* var. botrytis over *B*. *carinata* (*χ*^2^ = 11.63, df = 1, *P* < 0.01, N = 88), and *B*. *napus* over *B*. *carinata* (*χ*^2^ = 6.22, df = 1, *P* < 0.01, N = 85). Adults exhibited no significant preference between *B*. *oleracea* var. botrytis and *B*. *napus* (*χ*^2^ = 1.84, df = 1, *P* = NS, N = 106).

**Fig 2:**
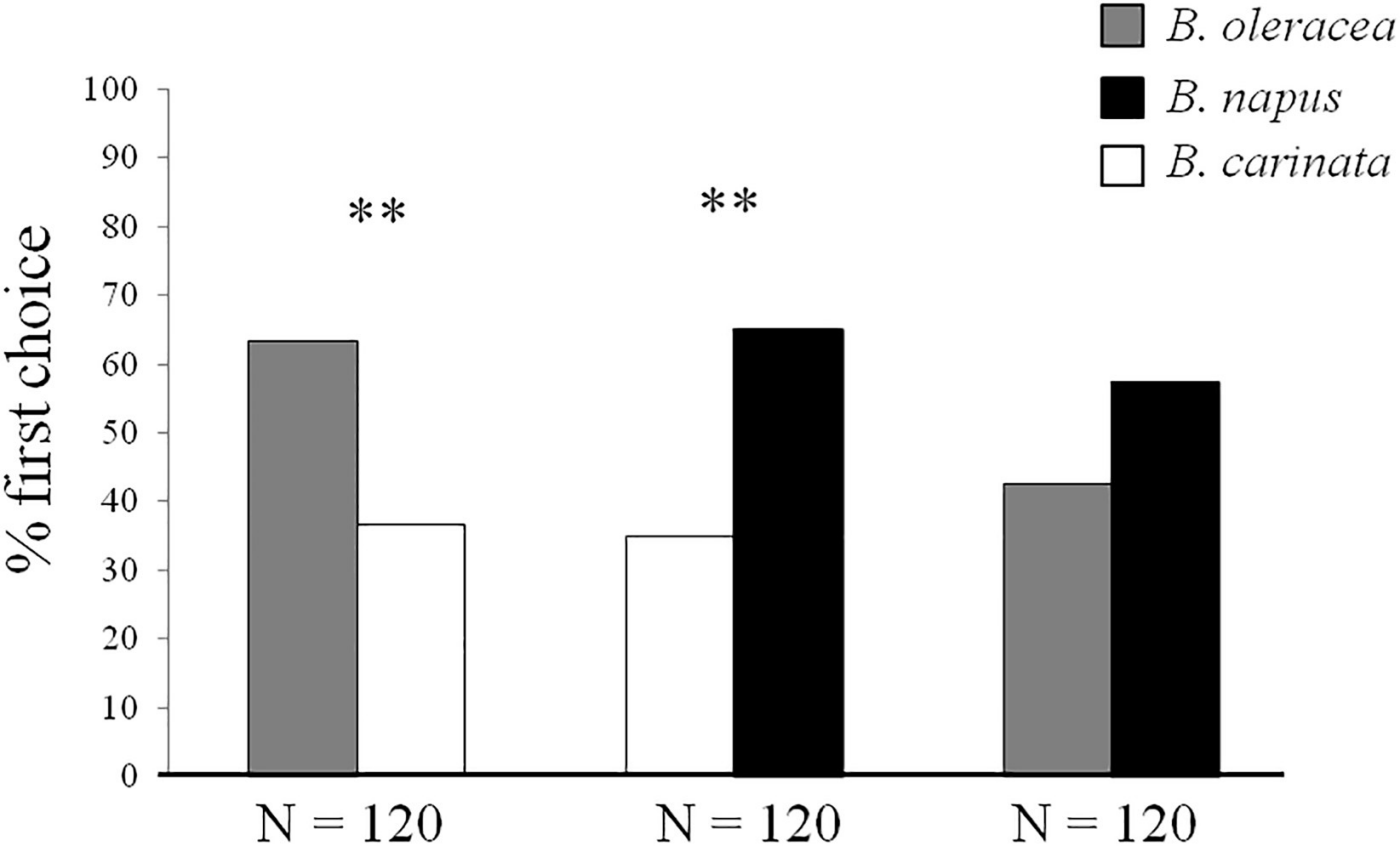
Y-shaped olfactometer bioassays. Host preference responses (% first choice) of *B*. *hilaris* adults to seedlings of *B*. *oleracea* var. botrytis, *B*. *carinata*, and *B*. *napus*. N = number of replicates; ** = P < 0.01; *χ*^2^.

In open vertical Y-shaped olfactometer bioassays (Fig 2), *B*. *hilaris* adults also were more strongly attracted to *B*. *oleracea* var. botrytis than *B*. *carinata* VOC (*χ*^2^ = 8.53, df = 1, *P* < 0.01 N = 120), and to *B*. *napus* than to *B*. *carinata* (*χ*^2^ = 10.80, df = 1, *P* < 0.01, N = 120). However, adult bugs were equally attracted to volatiles from *B*. *oleracea* var. botrytis and *B*. *napus* (*χ*^2^ = 2.27, df = 1, *P* = NS, N = 120).

### Bioassays with *B*. *oleracea* var. botrytis VOCs

**Fig 3:**
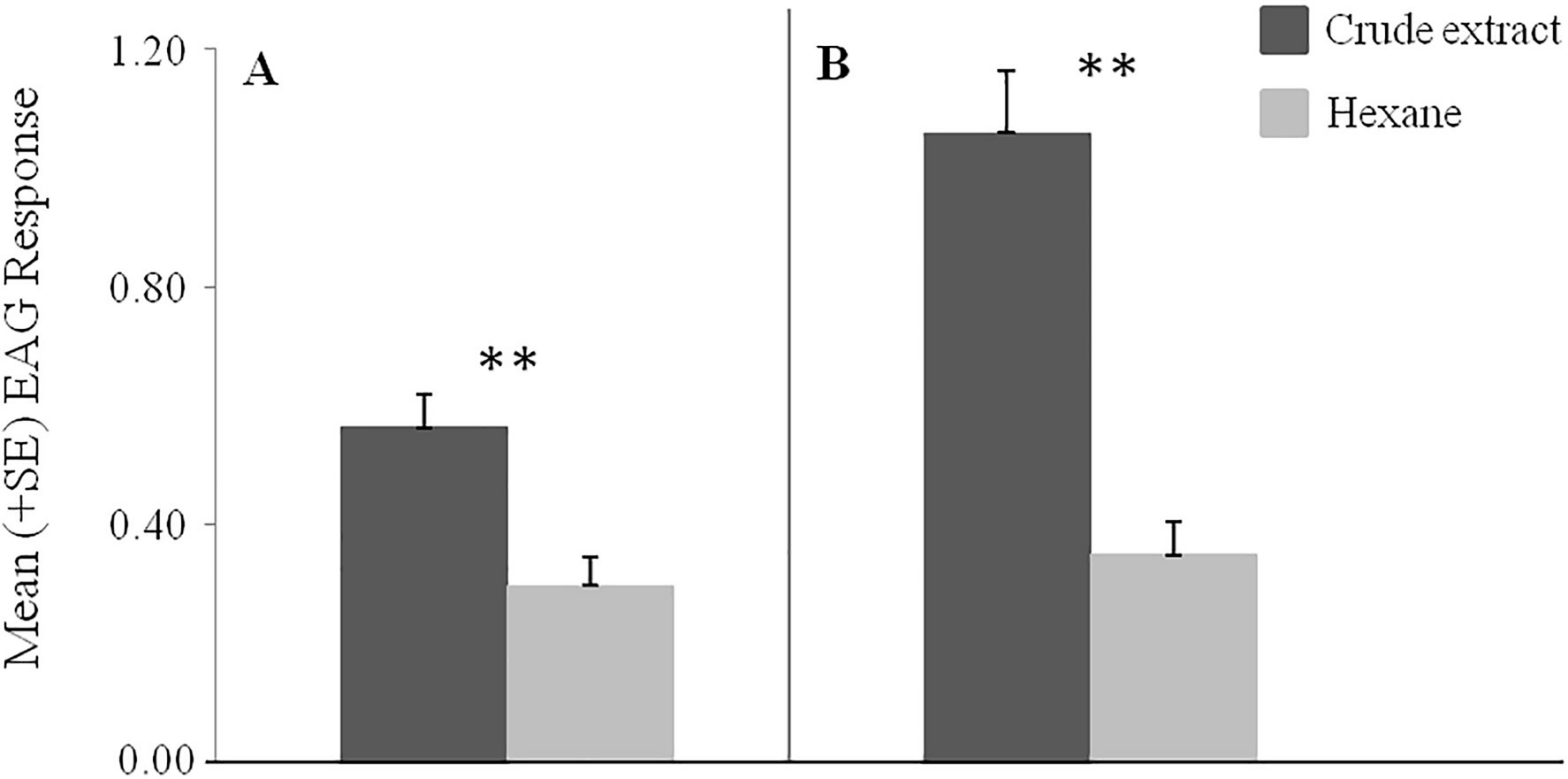
EAG bioassays to crude extracts of VOCs from *B*. *oleracea* var. botrytis seedlings. Electroantennogram responses (mV) (mean + SE) of antennae of *B*. *hilaris* 4^th^ and 5^th^ instar nymphs (N = 20) **(A)** and adults (N = 15) **(B)**; ** = P < 0.01; one-way ANOVA followed by Tukey’s test.

**Fig 4:**
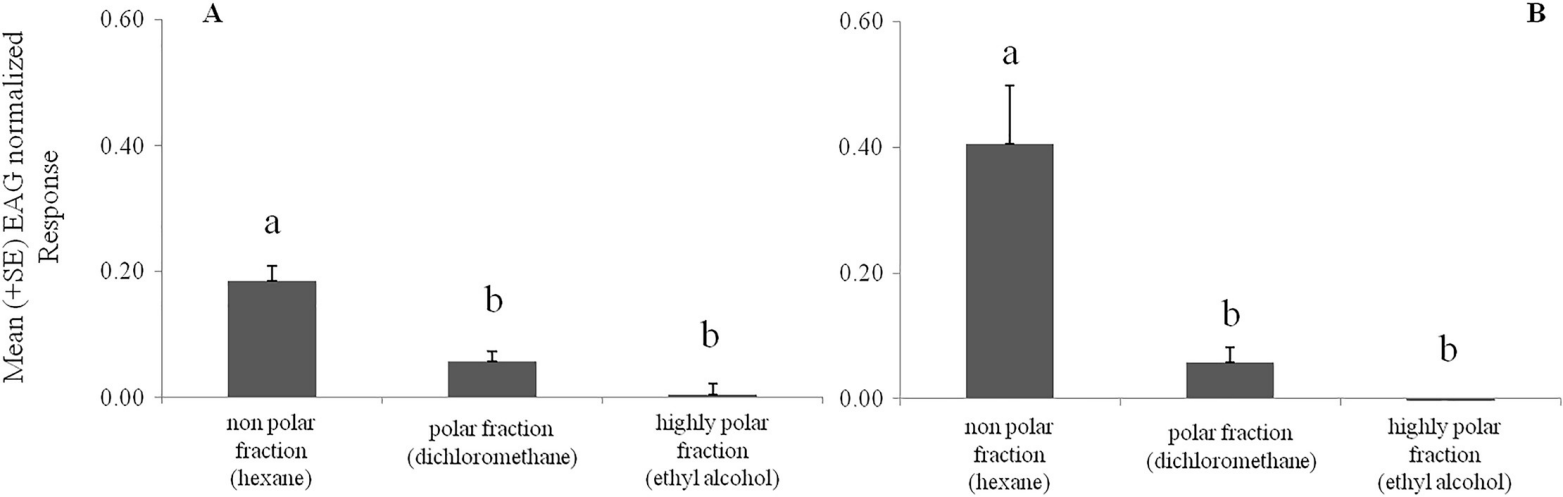
EAG bioassays to fractions of crude extracts of VOCs from *B*. *oleracea* var. botrytis seedlings. Electroantennogram responses (mV) (mean + SE) of *B*. *hilaris* 4^th^ and 5^th^ instar nymphs (N = 20) **(A)** and adults (N = 18) **(B)**; Different letters indicate that values differ statistically at P < 0.05; one-way ANOVA followed by Tukey’s test.

The crude VOC extract of *B*. *oleracea* var. botrytis elicited significantly higher EAG responses from the antennae of *B*. *hilaris* nymphs (*F* = 12.61, df = 1, *P* < 0.001, N = 20, ANOVA) (Fig 3A) and adults (*F* = 35.91, df = 1, *P* < 0.0001, N = 15, ANOVA) (Fig 3B) than the controls. Significantly different responses were also recorded from the different fractions of the VOC extract: the non-polar fraction elicited stronger responses than the intermediate and highly polar fractions from the antennae of 4^th^ and 5^th^ instar nymphs (*F* = 21.05, df = 2, *P* < 0.05, N = 20, ANOVA) (Fig 4A) and adults (*F* = 14.39, df = 2, *P* < 0.0001, N = 18, ANOVA) (Fig 4B).

**Fig 5:**
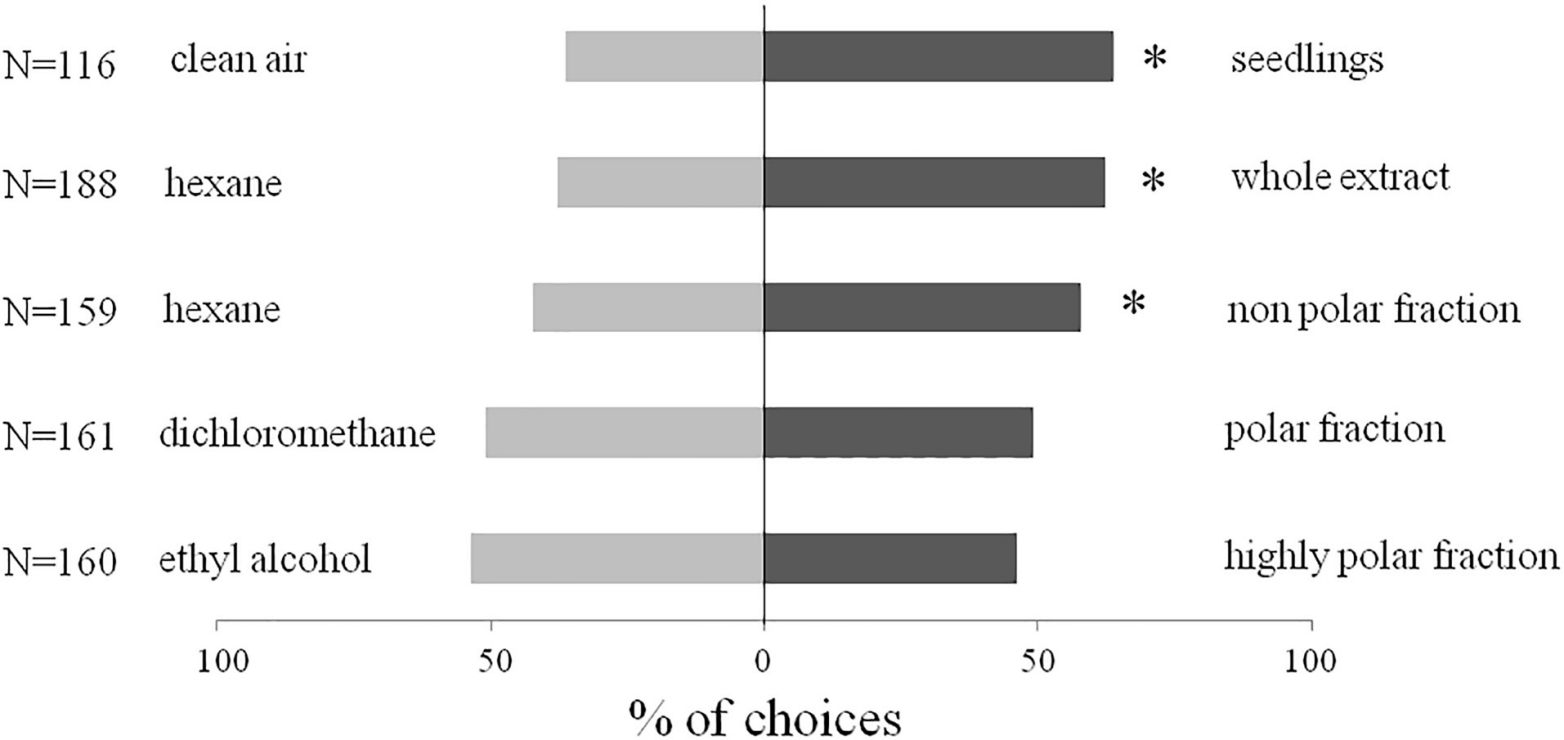
Behavioral bioassays with *B*. *hilaris* 4^th^ and 5^th^ instar nymphs. Responses (% first choice) to *B*. *oleracea* var. botrytis seedlings, to crude extracts of seedling VOCs, and to fractions therein, versus controls, in open vertical Y-shaped olfactometer bioassays. N = number of replicates; * = P < 0.05; *χ*^2^.

**Fig 6:**
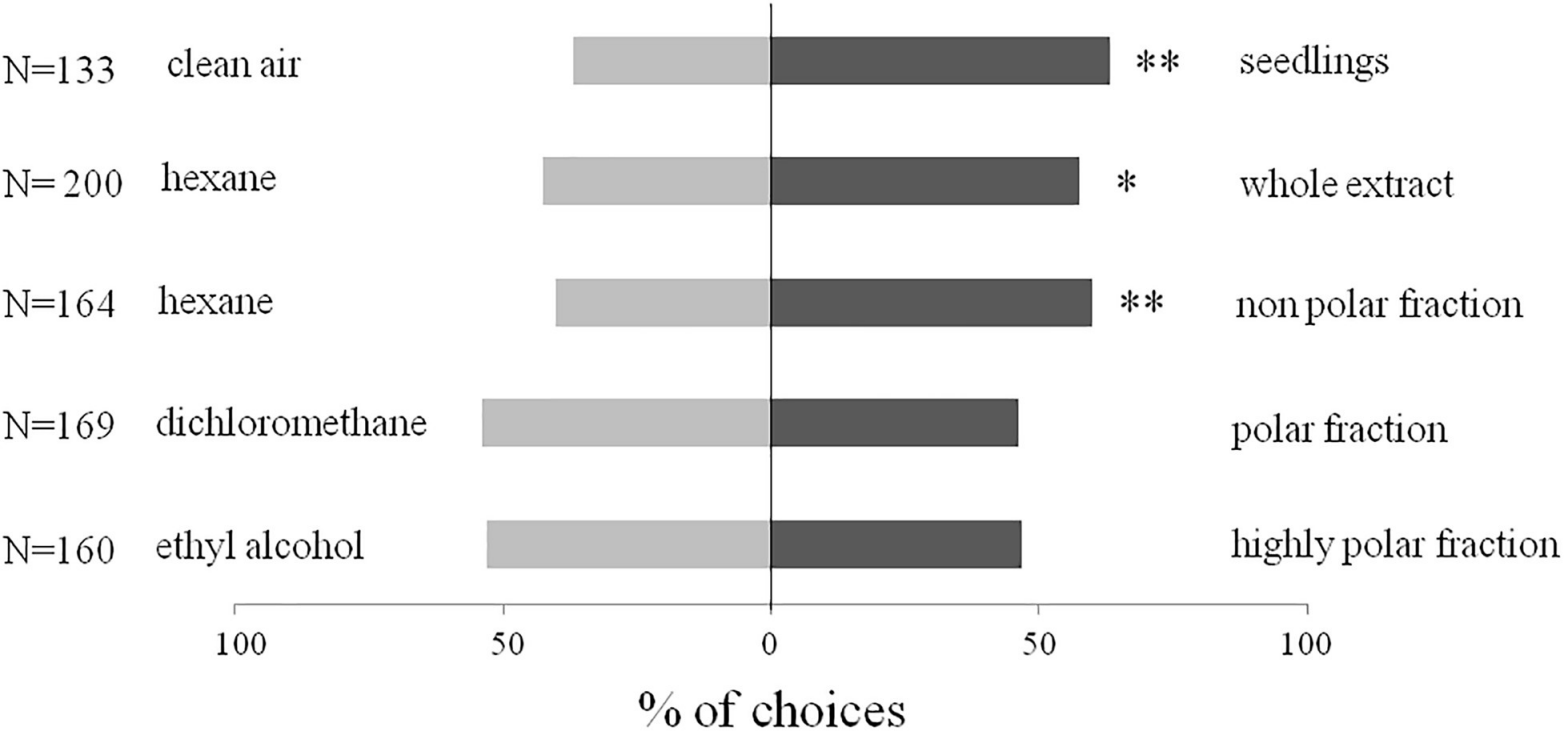
Behavioral bioassays with *B*. *hilaris* adults. Responses (% first choice) to *B*. *oleracea* var. botrytis seedlings, to crude extracts of seedling VOCs, and to fractions of the VOC extract, versus controls, in vertical Y-shaped olfactometer bioassays. N = number of replicates; * = P < 0.05, ** = P < 0.01; *χ*^2^.

Responses of *B*. *hilaris* adults and nymphs in open vertical Y-shaped olfactometer bioassays are shown in Figs 5 and 6. Seedlings of *B*. *oleracea* var. botrytis were significantly more attractive than controls (clean air) for 4^th^ and 5^th^ instar nymphs (*χ*^2^ = 8.82, df = 1, *P* < 0.05, N = 116) and for adults (*χ*^2^ = 9.21, df = 1, *P* < 0.01, N = 133). Similarly, the crude extract of VOCs from *B*. *oleracea* var. botrytis seedlings attracted both nymphs (*χ*^2^ = 11.25, df = 1, *P* = 0.01, N = 188) and adults (*χ*^2^ = 4.50, df = 1, *P* < 0.05, N = 200) in comparison to controls. When fractions of the crude extract were tested, bugs were significantly attracted only to the non-polar fraction eluted with hexane (hexane fraction versus solvent control, nymphs, *χ*^2^ = 3.93, df = 1, *P* < 0.05, N = 159, adults, *χ*^2^ = 6.24, df = 1, *P* < 0.01, N = 164). Neither of the more polar fractions was significantly more attractive than the solvent controls to nymphs (dichloromethane fraction, *χ*^2^ = 0.05, df = 1, *P* = NS, N = 161; ethyl alcohol fraction, *χ*^2^ = 0.90, df = 1, *P* = NS, N = 160) or adults (dichloromethane fraction, *χ*^2^ = 1, df = 1, *P* = NS, N = 169; ethyl alcohol fraction, *χ*^2^ = 0.62, df = 1, *P* = NS, N = 160).

### Chemical analysis

**Fig 7:**
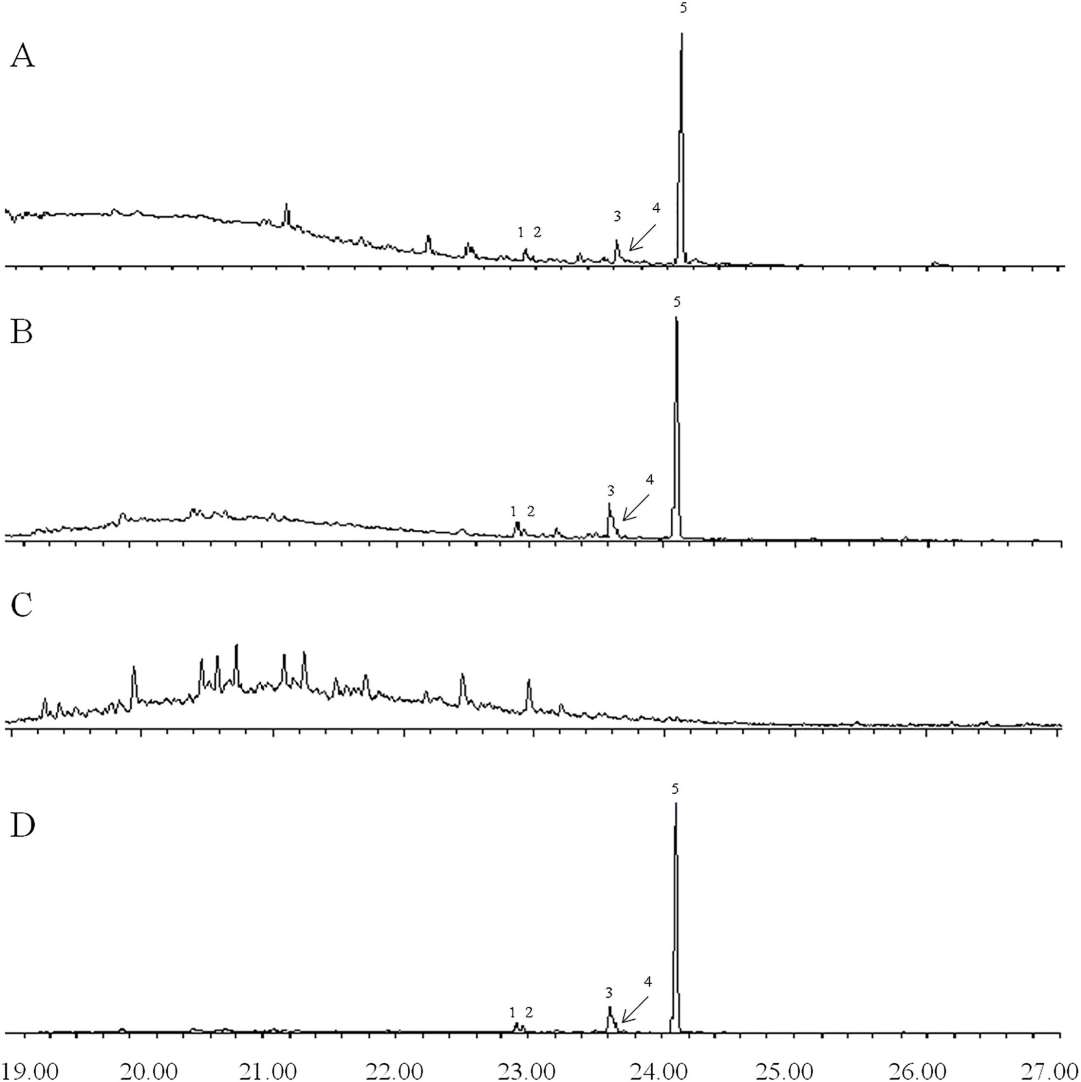
Representative gas chromatograms of VOCs from *B*. *oleracea* var. botrytis. (A), *B*. *napus* (B), *B*. *carinata* (C), and *B*. *oleracea* var. botrytis non-polar fraction (D). The peak numbers indicate the diterpene hydrocarbons detected in the analysis.

**Fig 8:**
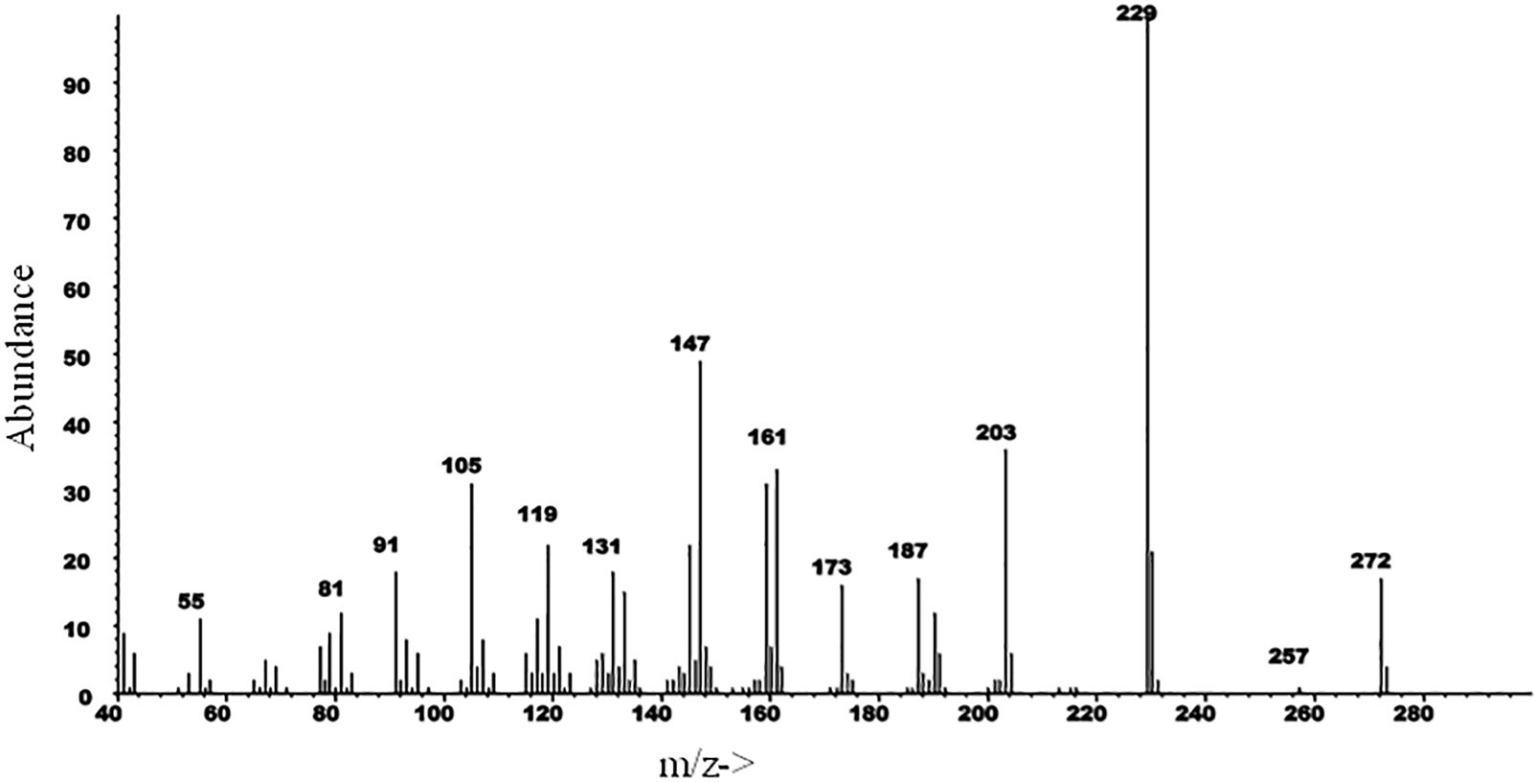
EI mass spectrum of the major compound in VOC extracts from *B*. *oleracea* var. botrytis.

**Fig 9:**
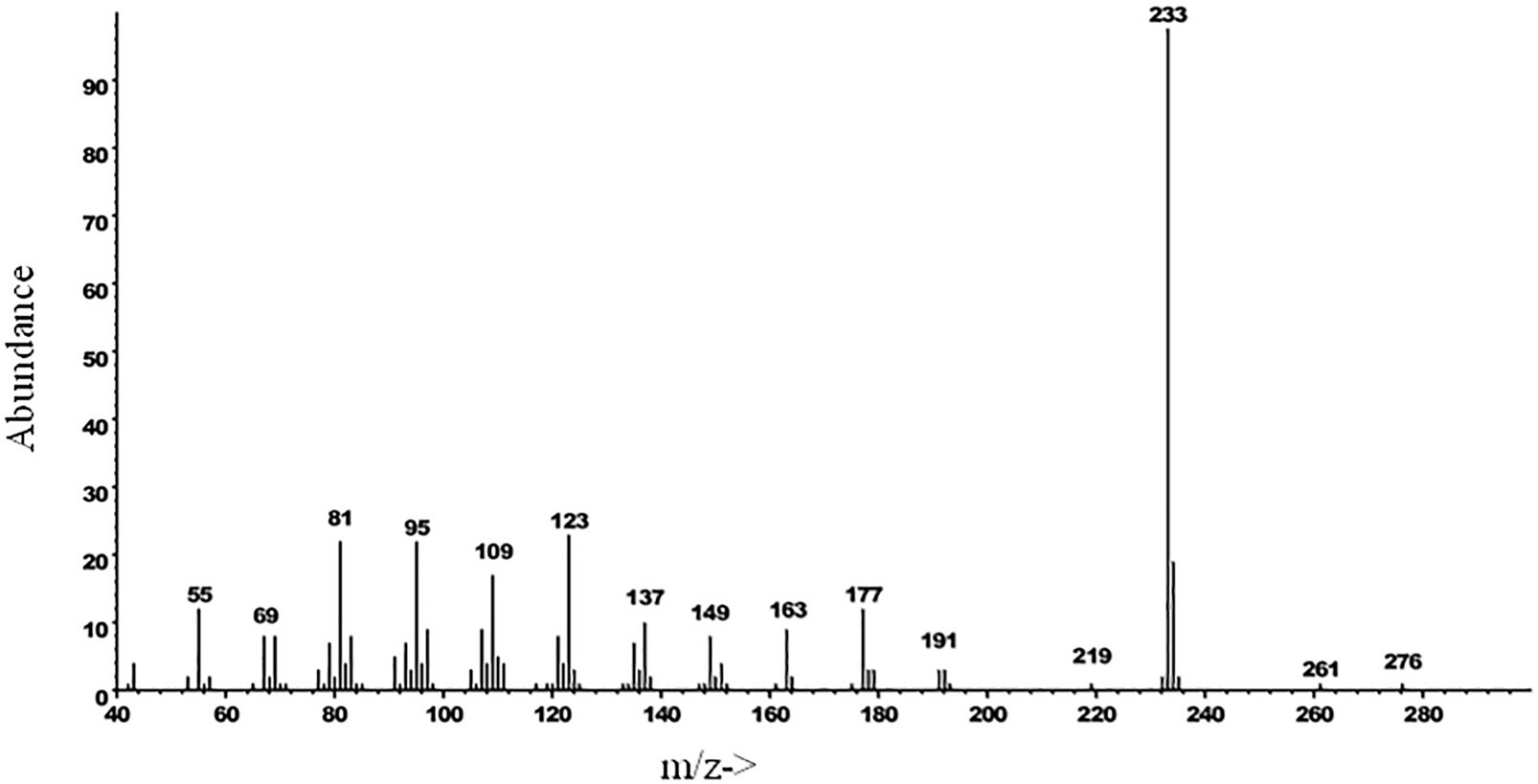
EI mass spectrum of the product from catalytic reduction of the major compound in VOC extracts from *B*. *oleracea* var. botrytis.

Representative chromatograms of VOCs collected from *B*. *oleracea* var. botrytis, *B*. *carinata*, and *B*. *napus* seedlings are shown in Fig 7. One major and four minor compounds were observed in the VOCs of *B*. *oleracea* var. botrytis and *B*. *napus* that were not detected in VOCs of *B*. *carinata*. A crude VOC extract of *B*. *oleracea* var. botrytis was fractionated by liquid chromatography on silica gel, eluting sequentially with hexane, dichloromethane, and ethanol, providing fractions of low, medium, and high polarity. The five compounds all eluted in the hexane fraction, indicating that they were hydrocarbons. The compounds were tentatively identified as diterpene hydrocarbons based on their molecular weights of 272 daltons, corresponding to molecular formulae of C_20_H_32_. The most abundant compound had a Kovats retention index of 1989 (DB5). Catalytic reduction with Pd on carbon produced a single compound with molecular weight 276, indicating the parent compound likely had two C = C double bonds, and hence, three rings to account for the three remaining sites of unsaturation. The mass spectra of the parent compound (Fig 8) and the product from catalytic reduction (Fig 9) were both dominated by a base peak from loss of 43 mass units, indicating the presence of an isopropyl group that was readily lost during fragmentation. The mass spectrum of the parent compound produced no reasonable matches with any structures in the NIST 11 Mass Spectral Database. Similarly, a search of all 996 structures in Chemical Abstracts with a molecular formula of C_20_H_32_, two double bonds, three rings, and an isopropyl group, failed to turn up any candidate structures whose mass spectra were a reasonable match to that of the major compound. The major component has now been isolated in microgram quantities by a combination of liquid and preparative gas chromatography and subjected to microbore NMR spectrometry, but the small amounts of material restricted the number and types of spectra that could be obtained. Thus, to date, we have not been able to arrive at a possible structure for the major (and minor) components in the extracts.

## Discussion

The results described above provide strong evidence that host plant VOCs are exploited by both nymphs and adults of *B*. *hilaris* in location and possibly acceptance of their host plants. The dual choice arena bioassay results indicated that some *Brassica* species are preferred over others, with *B*. *hilaris* adults preferentially orientating toward seedlings of *B*. *oleracea* var. botrytis and *B*. *napus* rather than *B*. *carinata*. These results were confirmed by the olfactometer bioassays, which suggested that attraction to preferred hosts is primarily mediated by olfactory rather than visual cues. Other recent studies with *B*. *hilaris* had demonstrated that this species was attracted to seedlings of several brassicaceous species, including arugula (*Eruca sativa* L.), turnip (*B*. *rapa* L. var. rapa), mizuna (*B*. *rapa* L. nipposinica), kale (*B*. *oleracea* L. acephala), choi (*Brassica rapa* L. var. chinensis), broccoli (*B*. *oleracea* L. var. italica), cauliflower (*B*. *oleracea* L. var. botrytis), and to a lesser extent sweet alyssum (*Lobularia maritima* L.), as well as non-brassicaceous seedlings of species such as lettuce (*Lactuca sativa* L.) [22, 23]. However, the present study is the first to show a direct relationship between host plant VOCs and painted bug host preference via the demonstrated attraction of painted bugs to extracts of VOCs. Analogous results have been observed with other pests of *Brassica* spp. plants [24]. Bioassays with fractions of the crude extract of *B*. *oleracea* var. botrytis VOCs then showed that the attraction of *B*. *hilaris* was mediated by one or more compounds contained in the non-polar fraction of the extract, consisting only of hydrocarbons.

In parallel with the behavioral trials, EAG recordings showed that the crude extract of volatiles from *B*. *oleracea* var. botrytis seedlings elicited significant responses from the antennae of *B*. *hilaris* adults and 4^th^-5^th^ instar nymphs. Further EAG recordings then localized the activity to the non-polar fraction of the VOC extract, with no significant responses being elicited by either of the more polar fractions.

GC-MS analyses of the crude VOC extracts showed that the VOCs emitted by *B*. *oleracea* and *B*. *napus* seedlings were quite similar, whereas the VOCs of *B*. *carinata* seedlings were markedly different. Surprisingly, the common green leaf volatiles and monoterpenes observed in other studies of *B*. *oleracea* var. botrytis volatiles using plants 4–5 weeks old or older [9, 25, 26] were not observed in the VOCs from seedlings. This result suggests that plants at the seedling stage, with only cotyledon leaves, emit VOCs quite different from older plants with true leaves. Rather, the VOCs from seedlings of *B*. *oleracea* var. botrytis and *B*. *napus* were dominated by a single diterpene hydrocarbon, with lesser amounts of several analogs. The mass spectra of these diterpenes did not match any spectra in the NIST 11 mass spectral database or any published spectra of diterpenes with three rings, two double bonds, and an isopropyl group, so they may represent novel compounds. Several diterpenes recently have been reported from the roots of the brassicaceous plant *Arabidopsis thaliana* L., but because of the difficulty in identifying the complex, often multicyclic structures of these types of compounds, only a few of these compounds were fully characterized [27, 28]. Because the diterpenes detected in this study constitute > 95% of the non-polar fraction of the VOCs of *B*. *oleracea* var. botrytis seedlings that attracted and elicited EAG responses from *B*. *hilaris* in our study, it seems likely that they are the key compounds exploited by *B*. *hilaris* in its strong attraction to seedlings of its host plants. However, at present, it is not possible to exclude the possibility that other hydrocarbons present in minor amounts in the non-polar fraction may be partly or wholly responsible for the activity seen.

The possibility that *B*. *hilaris* may exploit diterpenes for host location is interesting, given that these types of compounds are generally thought to contribute to plant chemical defenses [27, 29]. For example, the protective role of plant diterpenes in defense against biotic factors has been demonstrated by their antifeedant activity against *Pieris brassicae* L. [30] and *Heliothis armigera* Hübner, [31], and for antifungal properties toward several phytopathogenic fungi [32–34]. The presence of these defensive compounds in cotyledons, together with non-volatile compounds such as glucosinolates, may be explainable based on the importance of such tissues for the future development of the plant; according to optimal defense theory, the most valuable parts of a plant should also be the most heavily defended [35, 36]. However, insect specialists on brassicaceous plant species, such as *B*. *hilaris*, may have evolved adaptations to excrete or detoxify such defensive plant compounds [37, 38]. Taken one step further, this pest may have evolved to actually exploit this chemical defense as a cue to locate *Brassica* seedlings, analogous to other specialist herbivores which exploit their hosts’ defensive chemistry rather than being deterred by it, such as lygaeid bugs which sequester toxic cardenolides from their milkweed hosts [39], or flea beetles which sequester glucosinolates from their brassicaceous hosts [40]. In fact, *B*. *hilaris* may have further refined this strategy by evolving to find and exploit the particularly susceptible and nutrient-rich seedling stage of its hosts.

Several other studies on herbivore-plant interactions have elucidated the crucial role of such volatile secondary plant compounds that act as host location kairomones for herbivores [41–43]. In fact, the importance of these secondary plant substances as cues exploited for host plant selection was emphasized several decades ago by Fraenkel [44] as “*the very heart of agricultural entomology*”. However, in most cases, host plant location by phytophagous insects is likely determined by the perception of ubiquitous phytochemicals such as green leaf volatiles and monoterpenes which may be emitted from particular plant species in specific ratios [45, 46]. In contrast, in interactions between herbivores and brassicaceous host plants, including the *B*. *hilaris–B*. *oleracea* system, attraction to hosts is often mediated by the perception of relatively uncommon compounds which are specific to one or a small group of closely related host species [37, 47–49].

From an ecological viewpoint, the work described here provides fundamental knowledge about the chemical cues exploited by *B*. *hilaris* for host location. For practical purposes, this work may also represent an important step in the discovery of useful attractants for monitoring this pest, particularly because no effective attractants are currently available for this species. Alternatively, seedlings of highly attractive varieties could possibly be used as a trap crop, attracting *B*. *hilaris* away from the cash crop, and/or to specific areas for treatment with pesticides. Furthermore, the fact that some *Brassica* species, such as *B*. *carinata*, apparently lack these diterpenes may provide opportunities for developing hybrids, that do not emit these compounds, either by traditional plant breeding methods, or by genetic manipulation of the plants. Efforts to fully identify the major and minor diterpenes, using a variety of spectroscopic and microchemical techniques, are ongoing.

## Supporting information

S1 Table Host preference bioassays carried out in dual choice arena and Y-shaped olfactometer. (XLSX)

S2 Table Electroantennography. (XLSX)

S3 Table Y-shaped olfactometer bioassays with *B. hilaris* nymphs and adults using VOCs of *B. oleracea* var. botrytis seedlings, crude extracts and to fractions therein. (XLSX)
